# Liver Abscess: Think Outside the Box

**DOI:** 10.7759/cureus.86755

**Published:** 2025-06-25

**Authors:** Konstantina Charisi, Maria Terzaki, Athanasios Bangeas, Anna Taparkou, Eleni Papadimitriou, Erieta Karypidou, Dimitrios Kouroupis, Maria S SidiropouIou, Athina Pyrpasopoulou, Emmanuel Roilides, Evangelia Farmaki

**Affiliations:** 1 1st Department of Pediatrics, Ippokrateio General Hospital of Thessaloniki, Thessaloniki, GRC; 2 2nd Propedeutic Department of Internal Medicine, Ippokrateio General Hospital of Thessaloniki, Thessaloniki, GRC; 3 Department of Radiology, Ippokrateio General Hospital of Thessaloniki, Thessaloniki, GRC; 4 3rd Department of Pediatrics, Ippokrateio General Hospital of Thessaloniki, Thessaloniki, GRC

**Keywords:** chronic granulomatous disease, corticosteroids, dihydrorhodamine oxidation test, liver abscess, staphylococcus aureus

## Abstract

Liver abscesses are a relatively common pathology, especially in adults, and are usually secondary to intrabdominal infections. In patients with chronic granulomatous disease (CGD), a rare primary immune deficiency, they may represent the main or even first manifestation of the disease. Clinical suspicion should be raised especially in recurrent forms and when typical pathogens are implicated. We report the case of a 37-year-old male who presented febrile, with recurrence of a liver abscess caused by *Staphylococcus aureus*, and was treated with corticosteroids, besides targeted antibiotics and standard of care. Accurate diagnosis of the underlying medical condition is crucial to apply a more adequate treatment and ensure better short- and long-term prognosis.

## Introduction

Chronic granulomatous disease (CGD) is a rare primary immunodeficiency (one in every 200,000-250,000 live births), caused by mutations in any of the five subunits of the nicotinamide adenine dinucleotide phosphate (NADPH) oxidase complex. The host NADPH oxidase is essential in the first-line defense against pathogens. It oxidizes intracellular NADPH to reduce extracellular oxygen to produce superoxide anions that participate in pathogen killing [1]. Mutations affecting the structure and function of this enzyme compromise the respiratory burst in phagocytic leukocytes [2] and put the affected patients at increased risk for invasive bacterial and fungal infections. These may involve any organ but more eagerly affect the lymph nodes, liver, and lungs. Pathogens most frequently isolated from patients with CGD include, but are not restricted to, *Staphylococcus aureus, Aspergillus *spp, *Serratia *spp, *Nocardia *spp, and* Burkholderia cepacia *[3]. Common clinical manifestations are deep-seated infections, recurrent and multiple abscesses, recurrent pneumonia, and granulomatous lymphadenitis. Granuloma formation is central to the pathophysiology of the disease and develops from the long-lasting hyper-inflammation associated with the deficient protracted activation of the phagocytes. This, in turn, may lead to other complications, such as the development of inflammatory bowel disease, like colitis [4]. CGD is usually diagnosed in the first years of life; however, patients may become diagnosed much later in adulthood despite their suspicious history. Residual activity of the NADPH oxidase complex may account for the less remarkable clinical manifestations, infections. CGD is classified as X-linked and autosomal recessive, respectively. The X-linked form is significantly more frequent (~65% of cases), especially among male patients. The diagnostic test of choice is the dihydrorhodamine assay which can additionally detect gene mutation carriers and provide information on inheritance patterns [5]. Liver abscesses are a common presentation of the disease and are encountered in 30%-50% of the patients [6]. We present the case of a 37-year-old male who was diagnosed with CGD during investigation of recurrent liver abscesses.

## Case presentation

A 37-year-old male presented with high-grade fever (40.0^o^C) of 10-day duration and right upper quadrant abdominal pain. Medical history included a hospital admission for a liver abscess 10 years earlier, frequent febrile upper respiratory tract infections, and a prolonged hospitalization during childhood for fever of unknown origin with spontaneous remission. At the previous hospitalization for liver abscess, the patient was treated empirically with amoxicillin clavulanic acid for a period of three weeks with good clinical response. No follow-up visit was recorded in his medical notes. The patient did not receive any regular medications.

At presentation, the patient was febrile (38.6^o^C) but hemodynamically stable. Clinical examination was remarkable only for right upper quadrant abdominal tenderness. Laboratory tests and imaging were requested. Inflammatory markers were elevated (WBC 16,800 per μL (normal values, nv <10,000) and increased C-reactive protein (CRP) 253 mg/L, nv <5). Echographically, the liver was of normal size; an avascular hypoechoic lesion was detected along with cystic lesions with echogenic content and irregular borders. With the aid of CT scan, these right hepatic lobe lesions were characterized as abscesses measuring up to 8 cm.

**Figure 1:**
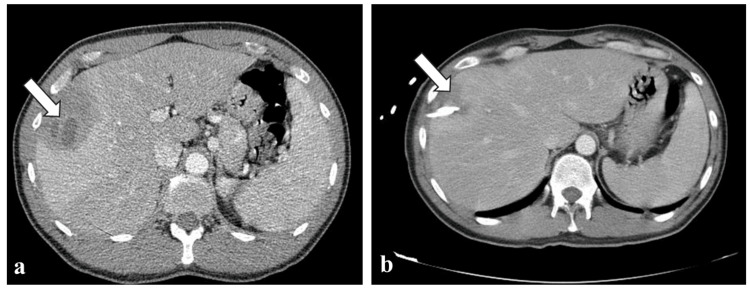
CT scan of the upper abdomen. CT scan of the upper abdomen revealing a septated non-homogeneous right hepatic lobe mass (arrow), compatible with a chronic abscess, with “cluster and double target sign”, defined as a thick walled multiloculated lesion (cluster sign) with central low attenuation fluid-filled component surrounded by a high attenuation inner rim and a low attenuation outer ring (double target sign) before (a) and after (b) CT-guided insertion of catheter and drainage.

A complete work-up for liver abscesses was requested. Microscopic stool examination and stool as well as blood cultures taken prior to the administration of antibiotics did not identify any pathogens. Serological tests for Echinococcus and Bartonella were negative. CT-guided drainage of the abscesses was performed, and the culture grew methicillin-sensitive *Staphylococcus aureus* (Figures 1a, 1b). The patient was treated with cloxacillin (2 g every four hours intravenously) with moderate reduction of inflammatory markers and persistence of fever (Table 1).

**Table 1:** Laboratory parameters at presentation (day 1) and during hospitalization; corticosteroids were initiated after day 14. WBC: white blood cells, Hb: hemoglobin, PLTs: platelets, SGOT: aspartate aminotransferase, SGPT: alanine aminotransferase, AlP: alkaline phosphatase, γgt: gamma-glutamyl transferase, LDH: lactate dehydrogenase, Ur: urea, SCr: serum creatinine, CRP: C-reactive protein, nv: normal values, U: units.

Lab parameter	Day 1 (admission)	Day 7	Day 14	Day 21	Day 28	Day 35	Day 40
WBC (10^3^/μL; nv 4.5-10.5)	15.3	13.1	9.7	14.0	12.5	10.2	11.0
Hb (g/dL; nv 11.5-14.5)	11.6	10.6	10.2	11.7	11.7	12.0	11.5
PLTs (10^3^/μL; nv 150-400)	341	489	575	830	432	358	449
SGOT (U/L; nv 10.0-37.0)	15	13	19	29	47	41	41
SGPT (U/L; nv 10.0-45.0)	36	19	18	91	99	116	107
AlP (U/L; nv 30.0-120.0)	102	77	104	131	122	121	120
γgt (U/L; nv <55)	91	66	58	98	74	74	107
LDH (U/L; nv <248)	136	125	349	197	161	190	261
Ur (mg/dL; nv 10.0-43.0)	22	27	24	59	40	28	37
SCr (mg/d; nv 0.8-1.25)	1.13	0.82	0.73	0.78	0.80	0.69	0.71
CRP (mg/L; nv<6)	257.3	197.9	64.1	10.3	31.6	50.0	72.2

**Figure 2:**
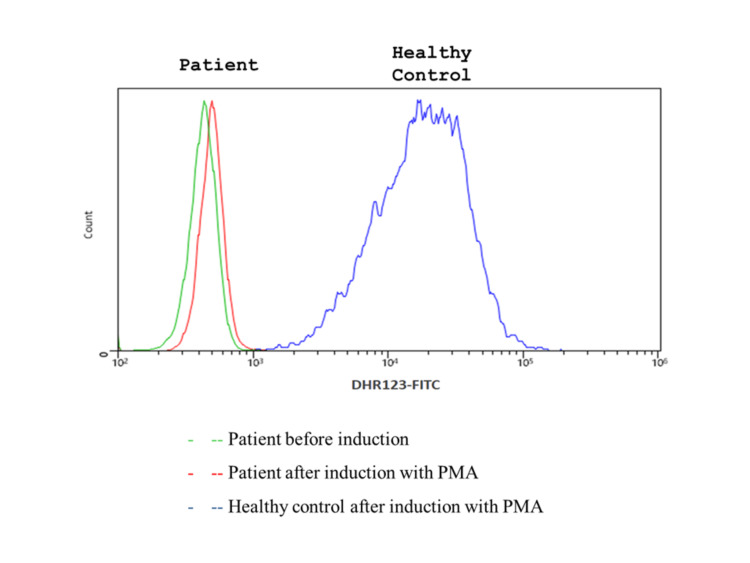
Patient and control fluorescence histograms obtained in the DHR123 assay by flow cytometry. Complete fluorescence shift was observed after phorbol myristate acetate (PMA) stimulation in the healthy control in comparison to the patient. No fluorescence is detected after induction in the patient’s sample indicating lack of oxidase activity in stimulated phagocytes.

Due to the patient’s history, the catalase positivity of the pathogen, and the persistence of symptoms, a dihydrorhodamine oxidation (DHR) test was performed. Neutrophil oxidative burst, tested with the DHR assay before and after stimulation of neutrophils with phorbol myristate acetate (PMA), repeatedly resulted in lack of DHR fluorescence, setting the diagnosis of CGD (Figure 2). The patient’s mother was tested for CGD with the same technique, ruling out an X-linked mode of transmission; his one-year-old male offspring’s phagocytic activity was also normal [7]. Genetic mutation confirmation was not performed, as diagnosis and the disease's mode of inheritance was set based on the assay used, and the molecular testing was inaccessible and associated with significant cost.

Corticosteroids were added to the continuous targeted antibiotic regimen (methylprednisolone at a dose of 0.5 mg/kg) on day 14 leading to rapid and sustained clinical, laboratory, and radiological improvement. The patient was discharged on long-term antibiotic treatment (completion of a three-month regimen with rifampicin 600 mg qd (once daily) and co-trimoxazole 960 mg td (three times daily)) and corticosteroid tapering and is regularly followed up being on antibacterial and antifungal prophylaxis (co-trimoxazole 960 mg qd and itraconazole 200 mg qd).

## Discussion

Patients with CGD are characterized by an impaired ability of the NADPH oxidase complex to generate superoxide anion, a function which is important for the effective microbial killing by the phagocytes. Patients with CGD are at increased risk of life-threatening infections with catalase-positive bacteria, fungi, and, rarely, mycobacteria and inflammatory complications such as colitis, myocardial or respiratory tract disease, and chorioretinitis [8]. The patient's diagnosis was missed during the previous hospitalization for liver abscess, which was most probably inadequately treated, thus paving the way for its recurrence. Recent reports suggest that corticosteroids appear to effectively halt aberrant inflammation and thus control refractory infections [9]. Outcome of liver abscesses in the context of CGD is significantly improved by the adjunct administration of corticosteroids [10,11]. However, comparison of corticosteroid therapy to traditional invasive treatments is still lacking [12].

Most patients are diagnosed by the age of five years. Still, the condition may remain undiagnosed until adulthood, mainly in the autosomal recessive forms and depending on the residual activity of the NADPH oxidase complex. Management of CGD requires aggressive management of infectious complications and lifelong antibacterial and antifungal prophylaxis. Immune system inducers, such as interferon-γ, may be beneficial. This combination of treatments can reduce severe infections from one per patient per year to almost one per patient per 10 years. Still, the only established curative treatment for CGD is hematopoietic cell transplantation with higher success rates in younger and disease-free patients [13].

## Conclusions

Liver abscesses are a common presentation of CGD and are encountered in 30%-50% of the patients. This case emphasizes the need to consider this rare and atypical diagnosis when investigating liver abscesses, an otherwise not uncommon clinical presentation among adults. In the case of our patient, key elements of the patient’s anamnesis and present history, such as the recurrence of symptomatology, slow clinical improvement, and the type of pathogen (catalase-positive) prompted us to test his oxidase activity in stimulated phagocytes. High clinical suspicion leading to early diagnosis remains the cornerstone for better treatment and outcome. So, remember, think of horses when you hear hoofbeats, but keep zebras in mind as well.
